# Cadherin-2 Is Required Cell Autonomously for Collective Migration of Facial Branchiomotor Neurons

**DOI:** 10.1371/journal.pone.0164433

**Published:** 2016-10-07

**Authors:** Jane K. Rebman, Kathryn E. Kirchoff, Gregory S. Walsh

**Affiliations:** Department of Biology, Virginia Commonwealth University, Richmond, Virginia, United States of America; National Institutes of Health, UNITED STATES

## Abstract

Collective migration depends on cell-cell interactions between neighbors that contribute to their overall directionality, yet the mechanisms that control the coordinated migration of neurons remains to be elucidated. During hindbrain development, facial branchiomotor neurons (FBMNs) undergo a stereotypic tangential caudal migration from their place of birth in rhombomere (r)4 to their final location in r6/7. FBMNs engage in collective cell migration that depends on neuron-to-neuron interactions to facilitate caudal directionality. Here, we demonstrate that Cadherin-2-mediated neuron-to-neuron adhesion is necessary for directional and collective migration of FBMNs. We generated stable transgenic zebrafish expressing dominant-negative Cadherin-2 (Cdh2ΔEC) driven by the *islet1* promoter. Cell-autonomous inactivation of Cadherin-2 function led to non-directional migration of FBMNs and a defect in caudal tangential migration. Additionally, mosaic analysis revealed that Cdh2ΔEC-expressing FBMNs are not influenced to migrate caudally by neighboring wild-type FBMNs due to a defect in collective cell migration. Taken together, our data suggest that Cadherin-2 plays an essential cell-autonomous role in mediating the collective migration of FBMNs.

## Introduction

Neuronal migration is a fundamental step in the assembly of neural circuits that control behavior. Neuron migration integrates multiple cellular and molecular events to coordinate movement from their birth place to their final destination. In contrast to radial migration in which neurons follow radial glial fibers, tangentially migrating neurons use interactions with other cell types to guide their movement. These interactions may be homotypic, in which a neuron relies on interactions with the same class of neurons, or heterotypic, in which interactions occur between other cell types in their environment to guide their trajectory. Accumulating evidence indicates that some neurons migrate as clusters, chains, or streams, suggesting that they are better described as collective migrations [1]. Collective cell migration, defined as the coordinated migration of cells, depends on cell-cell interactions between neighbors that contribute to their overall directionality[2–4]. The exact nature and importance of cell-cell interactions that lead to collective migration of neurons is unclear, but likely involves the function of cell adhesion molecules.

Cadherins, a family of calcium-dependent cell adhesion molecules, engage in homophilic binding to regulate cell-cell adhesiveness and promote adherens junction formation in stationary epithelial tissues. Cadherin-2 (N-cadherin; Cdh2) is broadly expressed throughout the developing nervous system and Cdh2-based cell-cell interactions are involved in various processes during neural development including tissue architecture, neuron migration, axon elongation, pathfinding and fasciculation, target recognition and synaptogenesis [5]. Inactivation of Cdh2 in mice and zebrafish results in severe neural tube formation defects [6,7], making it difficult to determine a role for Cdh2 in later developmental events. However, neuron-specific inactivation of Cdh2 impairs both pia-directed migration of cortical neurons along radial glial fibers by interfering with cell-substrate adhesion [8,9] and leads to impaired tangential migration of neurons from the medial ganglionic eminence to the cortex [10,11] as well as the chain migration of cerebellar granule cells [12], where directional movement is coordinated by Cdh2-based cell-cell contacts.

In this report, we use facial branchiomotor neurons (FBMNs) as a model system to study collective neuron migration. FBMNs are a subset of cranial branchiomotor neurons that are born in the ventral portion of rhomobomere 4 (r4) in the developing hindbrain. These neurons undergo a posterior tangential migration along the ventral portion of the hindbrain to r6 where they form the facial motor nucleus [13]. During migration, the facial motor axons are laid down behind the migrating neuronal cell bodies that exit the hindbrain at the level of r4 to innervate muscles derived from the second branchial arch [14].

The migration of FBMNs requires heterotypic cellular interactions with the surrounding neuroepithelial cells as well as homotypic interactions with other FBMNs to coordinate their caudal directionality. It is now established that components of the planar cell polarity (PCP) pathway function both non-cell-autonomously (in the environment), as well as cell-autonomously *within* FBMNs to control their caudal trajectory [15–24]. FBMNs, therefore, display a PCP-dependent mode of migration that requires an interaction with the planar-polarized neuroepithelial cells [22,23]. FBMNs also engage in collective cell migration since the migration of one FBMN can influence the migration of another neighboring FBMN [22]. Collective migration of FBMNs can be visualized in chimeric embryos generated by cell transplantation in which a wild-type FBMN can promote the caudal migration of a PCP-deficient FBMN [22]. Thus, the collective mode of migration promotes the caudal directionality of FBMNs in a PCP-independent manner that requires homotypic FBMN-to-FBMN interactions. The molecular nature of this cell-contact mediated collective neuron migration is not known.

Cadherin-2 is expressed in both migrating FBMNs and the surrounding neuroepithelial cells [6,25,26], and Cdh2 depletion has recently been reported to cause defects in FBMN migration [26,27]. Cdh-2 has been shown to promote neuroepithelial cell cohesion that is thought to limit dorsal movement and promote FBMN migration along the ventral aspect of the hindbrain [26]. Wanner and Prince reported that FBMN populations have a pioneer neuron, the first FBMN to migrate, that can direct follower neuron migration in the earliest phase of migration out of r4 and into r5 [27]. Indeed, laser ablation of the pioneer neuron or its trailing axon disrupts follower neuron migration [27]. Cdh2 knockdown decreases follower FBMN interactions with the trailing pioneer axon, indicating a role for Cdh2 in soma-to-axon interactions that promote caudal movement of follower FBMNs [27]. In both studies, a cell autonomous role for Cdh2 function within FBMNs was not directly tested.

Here, we test a role for Cadherin-2 as a cell adhesion molecule mediating the neuron-to-neuron interactions that drive the collective migration of FBMNs. Expression of dominant-negative Cdh2 specifically in FBMNs, and not the surrounding environment, results in a defect in caudal migration. This cell autonomous loss of Cdh2 function leads to random cell movements with resulting impairment in sustained caudal directional migration of FBMNs. Using mosaic analysis, we demonstrate that the impaired caudal migration of dominant-negative Cdh2-expressing FBMNs is not rescued by the presence of neighboring wild-type FBMNs due to a loss of collective migration. These results are consistent with a model in which Cadherin-2 is required cell-autonomously to drive neuron-to-neuron cell contact-mediated collective migration.

## Material and Methods

### Zebrafish Husbandry

Zebrafish were maintained following standard procedures and used in accordance with protocols approved by the VCU Institutional Animal Care and Use Committee. All zebrafish used in this study were raised and maintained in our fish facility. Embryos were collected and allowed to develop at 28.5°C to the required stage as described [28,29]. Transgenic lines were used as described: *Tg(islet1*:*GFP)rw0* [30].

### Plasmid DNA Constructs

The *zCrest1* enhancer of the *islet-1* (*isl1*) regulatory elements along with the minimal promoter from the *heat shock protein 70*, *like* (*hsp70l*) were PCR amplified, blunted and cloned into the EcoRV site in *pTolDest* (gift of Dr. Nathan Lawson) to make *pTol-isl1-hsp70l-DEST*. For simplicity, we will refer to the *isl1-hsp70l* promoter hereafter as the *isl1* promoter. *cdh2ΔEC* was PCR amplified from *pDONR-cdh2ΔEC*, a kind gift of Dr. William Harris [31] and fused in frame at the C-terminus with a 5 amino acid linker sequence with PCR amplified *mCherry* with Gibson assembly using primers to add *att* sites at the 5’ and 3’ ends of the assembled sequence. This sequence was then re-cloned into an entry vector to make *pME-cdh2ΔEC-mCherry*. Using Gateway cloning, this sequence was placed into *pTol-isl1-Dest* vector to generate *pTol-isl1*:*cdh2ΔEC-mCherry-Dest*. As a control, we also generated *pTol-isl1*:*mCherry-Dest*.

### Generation of *Tg(isl1*:*cdh2ΔEC-mCherry)* transgenic lines

Capped mRNA for Tol2 *transposase* was in vitro transcribed using the mMESSAGE mMachine kit (Ambion). DNA encoding each plasmid (50 ng/uL) was co-injected with Tol2 *transposase* mRNA (50 ng/uL) into *Tg(isl1*:*GFP)* embryos at the 1 cell stage. Founder (F0) embryos were screened for mosaic mCherry expression in cranial motorneurons at 24 and 48 hpf. F0 embryos that were doubly transgenic, displaying both GFP and mCherry expression, were raised to adulthood. Germline transgenic founders were identified by screening F1 progeny for GFP and mCherry fluorescence. Two founders (*vc23*, *vc25*) were isolated, mated with wild-type *Tg(isl1*:*GFP)* fish, and their GFP- and mCherry-positive progeny were raised to adulthood. To generate embryos containing two copies of the inserted transgene, F1 adults were incrossed and the F2 progeny displaying the brightest mCherry expression were raised to adulthood. To validate that these F2 transgenic fish were homozygous for the *isl1*:*cdh2ΔEC-mCherry* transgene, each F2 adult was crossed with wild-type *Tg(isl1*:*GFP)* fish, and the progeny were screened for the predicted expression of Cdh2ΔEC-mCherry in all embryos.

### Immunohistochemistry

The following primary antibodies were used. GFP: Mouse anti-GFP (DSHB; 1:100), Rabbit anti-GFP (Torrey Pines; 1:1000), Mouse anti-mCherry (NOVUS; 1:250). Embryos were manually dechorinated and fixed in 4% paraformaldehyde at 4°C overnight, washed in PBST (1× PBS with 0.25% Triton X-100), permeabilized with acetone and incubated for one hour at room temperature in blocking solution (PBST + 4% Goat Serum + 4% BSA). Embryos were then incubated in the corresponding primary antibody and diluted in blocking solution at 4°C overnight. They were then washed in PBST and incubated overnight at 4°C in secondary antibodies. The following secondary antibodies were used at a concentration of 1:200: Alexa Fluor 488 Goat anti-mouse IgG (H+L) (A11029, Life Technologies), Alexa Fluor 488 Goat anti-Rabbit IgG (H+L) (A11034, Life Technologies). Alexa Fluor 568 Goat anti-mouse IgG (H+L) (A11031, Life Technologies). Alexa Fluor 568 Goat anti-rabbit IgG (H+L) (A11079, Life Technologies). For some embryos, DAPI and Alexa Fluor 488-phalloidin staining was added to the secondary antibody solution. Embryos were washed in PBST and sequentially dehydrated in 25%, 50%, and 75% glycerol in 1× PBS. Yolks were removed using sharpened tungsten wire and embryos were flat mounted on coverslips and surrounded with 75% glycerol medium.

### Microscopy and timelapse imaging

Light microscope pictures were obtained on a Zeiss V8 stereomicroscope equipped with an IcC1 camera. Confocal images of immunostained embryos were obtained on an inverted Zeiss Spinning Disk Laser Confocal Observer Z1 using a Zeiss Plan-Apochromat 63X/1.2 W objective, or a Nikon C2 point scanning confocal microscope 40X/1.2 W objective for 48 hpf embryos. For analysis of FBMN motility, embryos were manually dechorionated and mounted in 1.2% low-melting point agarose on the coverslip of a glass bottom dish (Fluorodish; World Precision Instruments). Timelapse imaging was performed at 28.5°C using a heated stage insert. Time-lapse multiple focal plane images were obtained, with each z-stack collected every five minutes for a minimum of one hour. The acquired z-stacks were exported and analyzed using AR-Elements (Nikon) software. Embryo drift was corrected using ND alignment function. For movement analysis of FBMNs, each individual FBMN was manually traced, and the distance between centroids was tracked over multiple frames using AR-Elements software. Cell migration trajectories and instantaneous speed were measured and graphed using Prism Graphpad software.

### Heat shock

Fish water was preheated at 37°C. At 6 hpf, embryos were transferred to microcentrifuge tubes containing warmed fish water and placed in the 37°C water bath for 30 minutes. After 30 minutes, embryos were returned to petri dishes containing room temperature fish water and returned to the 28.5°C incubater. Embryos received 2 successive heat shocks spaced 30 minutes apart.

## Results

### Generation of *Tg(isl1*:*cdh2ΔEC-mCherry)* transgenic fish

**Fig 1:**
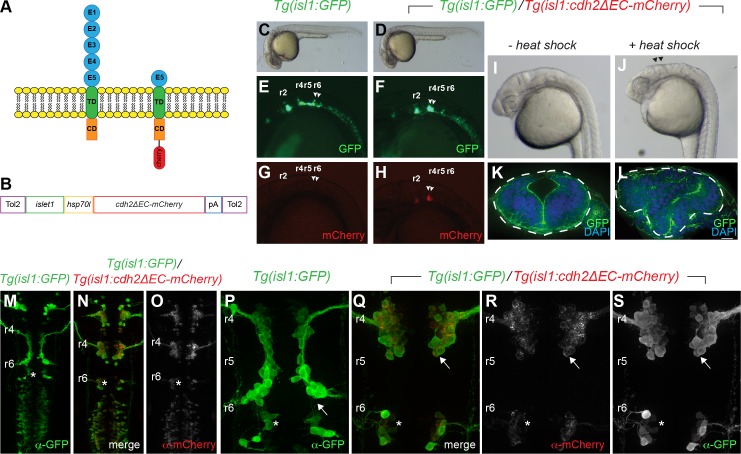
Generation of stable transgenic fish expressing dominant-negative Cadherin-2 in cranial branchiomotor neurons. (A) Schematic representation of full length Cadherin-2 (N-cadherin) and extracellular domain-deleted Cadherin-2 (Cdh2ΔEC) fused with mCherry that functions as a dominant-negative protein. E1-E5 cadherin ectodomains; TD, transmembrane domain; CD, cytoplasmic domain. (B). Schematic representation of plasmid used to generate stable transgenic fish expressing *cdh2ΔEC-mCherry* driven by the *zCrest1* enhancer element of the *islet1* promoter (*islet1*) upstream of the minimal promoter (*hsp70l*). (C,D) Photographs of wild-type *Tg(isl1*:*GFP)* and *Tg(isl1*:*GFP)/Tg(isl1*:*cdh2ΔEC-mCherry)vc25* embryos at 24hpf, showing normal morphology. (E-H) Lateral images at 24 hpf of the green and red channels showing GFP-expressing and Cdh2ΔEC-mCherry-expressing cranial branchiomotor neurons in *Tg(isl1*:*GFP)* fish and *Tg(isl1*:*cdh2ΔEC-mCherry)* transgenic fish. Arrowheads point to FBMNs. (I,J) Lateral images of *Tg(isl1*:*GFP)/Tg(isl1*:*cdh2ΔEC-mCherry)vc25* embryos at 24hpf with and without heat shock. Arrowheads denote defects at midbrain-hindbrain region after heat shock. (K,L) Cross sections through hindbrain neuroepithelium of *Tg(isl1*:*GFP)/Tg(isl1*:*cdh2ΔEC-mCherry)vc25* embryos before and after heat shock labeled with DAPI and Alexa-488-phalliodin. Dotted lines outline the neural tube. (M-S) Confocal micrographs of immunostained embryos showing low-magnification (M-O) and high magnification (P-S) dorsal views of wild-type *Tg(isl1*:*GFP)* (M,P) and *Tg(isl1*:*GFP)/Tg(isl1*:*cdh2ΔEC-mCherry)vc25* embryos (N,O,Q-S) at 26 hpf. Embryos were labeled with α-GFP and α-mCherry showing that Cdh2ΔEC-mCherry is only expressed in cranial branchiomotor neurons and not the surrounding neuroepithelium. Arrow points to the abnormal position of FBMNs in *Tg(isl1*:*cdh2ΔEC-mCherry)vc25* embryos. Rhomobomeres (r2-r6) are indicated. White asterisk denotes r6-derived PLL efferent neurons, which differ from r4-derived FBMN populations.

As *cdh2* is expressed in both FBMNs and the surrounding neuroepithelial cells [6,26], we sought to determine whether *cdh2* is required cell-autonomously within FBMNs for their migration. Previous studies, however, have shown that disrupting *cdh2* function in early development results in severe defects in neural tube formation [6,25]. Moreover, chimeric analysis has demonstrated that *cdh2* mutant cells are incapable of contributing to the ventral neural tube due to adhesive differences [6]. To bypass this, we expressed a dominant-negative form of zebrafish Cdh2, which has a deletion of the cadherin ectodomains 1–4 (Cdh2ΔEC), specifically in FBMNs using the *islet-1* (*isl1*) *zCREST1* enhancer upstream of a heat shock 70-like (*hsp70l*) minimal promoter (hereafter referred to as *isl1* promoter) [30,32] (Fig 1A). Work in multiple systems has demonstrated that overexpression of this dominant-negative form of Cdh2 results in a non-adhesive phenotype [31,33,34].

**Fig 2:**
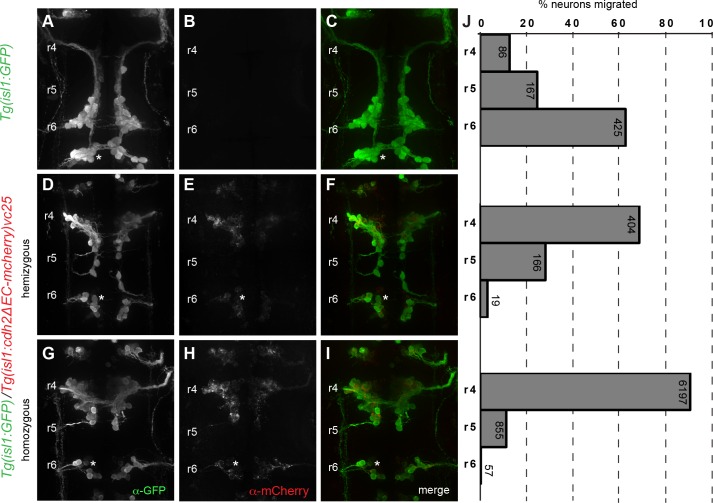
Cadherin-2 is required cell autonomously for caudal migration of FBMNs. (A-I) Whole-mount immunocytochemistry showing dorsal views of *Tg(isl1*:*GFP)* (A-C) and *Tg(isl1*:*cdh2ΔEC-mCherry)vc25* transgenic embryos (D-I) at 38 hpf embryos. Embryos are labeled with α-GFP (green) (A,D,G) and α-mCherry (red) (B,E,H) antibodies. (A-C) Wild-type *Tg(isl1*:*GFP)* embryos with FBMNs fully migrated into r6. (D-I) Defective caudal migration of FBMNs in *Tg(isl1*:*GFP)/Tg(isl1*:*cdh2ΔEC-mCherry)vc25* embryos carrying one copy of the transgene (hemizygous) or two copies (homozygous). (J) Histograms indicate the percent of FBMNS at 38 hpf that failed to migrate (r4), migrated partially (r5), or migrated fully (r6). Each histogram corresponds to the genetic condition in the image to its left and numbers indicate the number of FBMNs counted. White asterisk denotes r6-derived PLL efferent neurons, which differ from r4-derived FBMN populations.

Using the Tol2 transposition system [35], we were able to efficiently generate independent lines of *Tg(isl1*:*cdh2ΔEC-mCherry*) transgenic fish (Fig 1B). Two founders (*vc23*, *vc25*) were isolated that produced F1 progeny that showed expression of Cdh2ΔEC-mCherry in cranial branchiomotor neurons (CBMNs) (Fig 1C–1H). These founders were mated with wild-type *Tg(isl1*:*GFP)* fish, and their GFP-positive, mCherry-positive offspring were raised to adulthood. Adult F1 embryos were raised to sexual maturity and outbred to wild-type *Tg(isl1*:*GFP)* fish. Transgenic F2 progeny were generated at approximately 50% (47–53%, *n* > 200 per line), indicating a single insertion site in each founder, and a typical Mendelian inheritance pattern. We observed that the level of transgene expression varied between lines, with line *Tg(isl1*:*cdh2ΔEC-mCherry)vc23* (referred to as *vc23Tg*) having the lowest and line *Tg(isl1*:*cdh2ΔEC-mCherry)vc25* (referred to as *vc25Tg*) having the strongest expression. Following an incross of F2 transgenic adults, we also observed that the level of transgene expression was higher in F3 embryos with two copies of the transgene (homozygous) compared to F3 hemizygous transgene carriers (Fig 2E and 2H and S1 Fig). Despite expression of dominant-negative Cdh2 in CBMNs, these embryos were viable and fertile.

To confirm that the transgene functioned properly to inactivate Cadherin-2 function, we made use of the minimal promoter from the *heat shock protein 70*, *like* (*hsp70l*) in our transgenic construct. Under normal physiological conditions, this transgene drives tissue-specific expression in CBMNs due to the function of the *isl1* enhancer but can be used to drive expression in all tissues following heat shock. We reasoned that expression of *cdh2ΔEC-mCherry* in all tissues should phenocopy a *cdh2* mutant. We therefore subjected *vc25Tg* embryos to heat shock at shield stage to coincide transgene activation with the end of gastrulation but before the onset of neurulation. Examination of hindbrain morphology of *vc25Tg* embryos at 24 hours post fertilization (hpf) without heat shock revealed normal hindbrain architecture both in lateral views and in cross-sections (Fig 1I and 1K). Following heat shock, *vc25Tg* embryos displayed incomplete fusion of the dorsal neural tube with a mushroom-shaped hindbrain similar to that reported for *cdh2/parachute* mutants [6,25](Fig 1J and 1L). These results validate the efficacy of our transgene to inactivate Cdh2 function and indicates that overexpression of our transgene does not cause significant off-target phenotypic effects.

We examined transgenic lines for temporal and spatial expression of the *Tg(isl1*:*cdh2ΔEC-mCherry)* transgene. Both *vc23Tg* and *vc25Tg* transgenic lines displayed expression in CBMNs, consistent with previous reports using the z*CREST1* enhancer of the *islet1* regulatory elements [32] (Fig 1C–1H and Fig 1M–1P). Expression of the *cdh2ΔEC-mCherry* transgene was first detectable in trigeminal and facial branchiomotor neurons around 17 hpf, approximately one hour after they are born at 16 hpf. By 24 hpf, in addition to trigeminal neurons and FBMNs, *cdh2ΔEC-mCherry* transgene expression can be detected in most CBMN populations, including oculomotor, trochlear, r4-derived otic and lateral line efferents (Ole), r6-derived posterior lateral line (PLL) efferents, and vagal neurons (Fig 1O and 1R). In *Tg(isl1*:*cdh2ΔEC-mCherry)* fish, expression of Cdh2ΔEC-mCherry is apparent in FBMNs but was not detectable in neuroepithelial cells in the hindbrain (Fig 1O and 1R). Image analysis revealed an almost perfect overlap of *Tg(isl1*:*cdh2ΔEC-mCherry)* expression with *Tg(isl1*:*GFP)* expression in FBMNs (99.4%, *n* = 800 neurons, 7 embryos). These stable transgenic lines allowed us to examine the cell-autonomous role of Cdh2 in FBMN migration.

### Cadherin-2 acts autonomously to control caudal tangential migration of FBMNs

To determine whether Cdh2 is required cell-autonomously for neuron migration, we first examined the caudal migration of FBMNs in *Tg(isl1*:*cdh2ΔEC-mCherry)vc25* embryos at 24 hpf. In wild-type *Tg(isl1*:*GFP)* embryos, FBMNs are in the process of migrating caudally from r4 to r6 in wild-type (Fig 1M and 1P). In contrast, FBMNs in *Tg(isl1*:*cdh2ΔEC-mCherry)vc25* are specified correctly in r4 but have largely failed to exit r4 by 24 hpf (Fig 1N, 1O and 1Q–1S). We quantified the number of FBMNs migrating in *Tg(isl1*:*cdh2ΔEC-mCherry)vc25* embryos at 38 hpf, a timepoint in which migration is largely complete. In wild-type *Tg(isl1*:*GFP)* embryos, FBMNs have migrated properly to r6 (*n* = 678, 6 embryos) (Fig 2A–2C). In embryos hemizygous for either *vc25Tg* or *vc23Tg* transgene, FBMNs exhibit a severe defect in their caudal migration out of r4. In *vc25Tg* hemizygotes, we found that most FBMNs (68.5%, *n* = 589, 6 embryos) fail to exit r4, with a subset that migrates sparingly into r5 (28.2%), and only a small proportion of FBMNs (3.2%) that migrate into r6 (Fig 2D–2F). Embryos hemizygous for the *vc23Tg* transgene also display a defect in caudal migration, albeit less severe than that seen in *vc25Tg* embryos (S1 Fig). The severity of the migration phenotype correlated with the level of transgene expression between the two lines (Fig 2E and S1 Fig). To determine whether transgene dosage would affect phenotype severity, we analyzed embryos carrying two copies (homozygous) of the dominant-negative Cdh2 transgene. In both *vc25Tg* and *vc23Tg* homozygous embryos, the defect in caudal migration was more severe than in embryos hemizygous for the transgene (Fig 2G–2I and S1 Fig). In homozygous *vc25Tg* embryos that exhibit the highest level of transgene expression, we found that almost all FBMNs (87.1%, *n* = 7109 neurons, 56 embryos) failed to exit r4 (Fig 2G–2I). The correlation between phenotype severity and transgene expression underscores the importance of expression levels when using a dominant-negative approach.

Although the vast majority of FBMNs remain in r4 in homozygous *vc25Tg* embryos, we consistently observe a small proportion of FBMNs (12%, 855/7109 neurons, 56 embryos) that migrate caudally out of r4 and into r5 but not further. This suggests that FBMNs retain limited capacity for caudal movement in the absence of Cdh2 function.

We infrequently observed a small number of FBMNs that successfully traversed into r6 (0.8%; 57/7109 neurons, 56 embryos). These escaper FBMNs that migrate into r6 are characterized by the presence of a trailing axon that extends back towards r4 distinguishing them from r6-derived PLL efferents (marked by asterisk)(Fig 2G–2I and S2 Fig). We quantified the number and distribution of these escaper neurons per embryo. We found that many homozygous *vc25Tg* embryos (41%, *n* = 56 embryos) had no escaper FBMNs at all (S2 Fig). If present, escaper FBMNs were often only on one side of the embryo as one or two cells (45%, *n* = 56 embryos) (S2 Fig). We only found escapers on both sides of the embryo in a minority of homozygous *vc25Tg* embryos, either as one neuron on each side (5%, *n* = 56 embryos), or one neuron on one side and two on the other (9%, *n* = 56 embryos). Using image analysis, we verified that escaper FBMNs in r6 were Cdh2ΔEC-mCherry-positive (S2 Fig).

To ensure that this defect was not due to a delay in migration, we quantified the number of FBMNs migrating in wild-type (*n* = 8 embryos) and homozygous embryos *vc25TG* at 48 hpf, a timepoint when FBMN migration is complete. Again, we found that almost all FBMNs (89.8%, 1314/1464 neurons, 11 embryos) failed to exit r4 with no increase in the percentage of escapers that reached r6 at this later timepoint in homozygous *vc25Tg* embryos, suggesting that this defect is not due to delayed migration (S3 Fig). Taken together, our results demonstrate that Cdh2 function is required cell-autonomously within FBMNs for their proper efficient caudal migration.

### Cdh2 mediates directionality of FBMN migration

**Fig 3:**
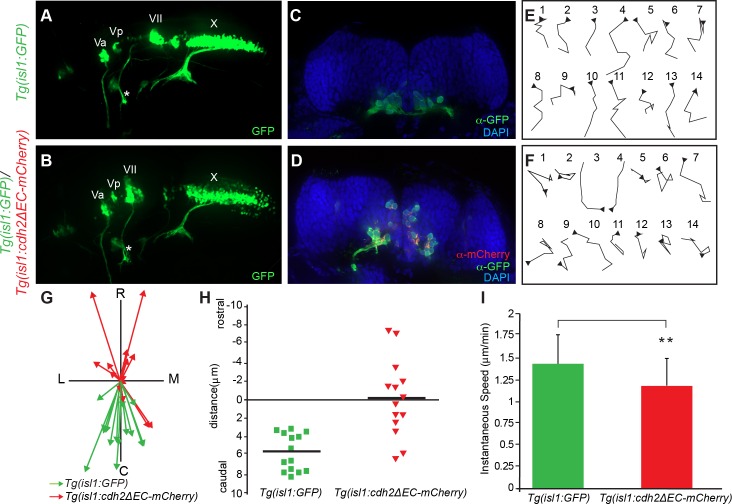
Inactivation of Cadherin-2 affects directionality of FBMN migration. (A,B) Live imaging of lateral views of the hindbrain showing the positioning of CBMNs and their peripheral axonal projections at 48 hpf. (A) In wild-type *Tg(isl1*:*GFP)* embryos, FBMNs (VII) migration is complete and their axons (asterisk) project into the second branchial arch. (B) In T*g(isl1*:*GFP)/Tg(isl1*:*cdh2ΔEC-mCherry)vc25* embryos, the facial axons (asterisk) and trigeminal (Va,Vp), and vagus (X) axons can be seen to project normally. However, the FBMNs (VII) remain in r4 and lie in an abnormal dorsal position. (C,D) Coronal sections of 48hpf hindbrain from *Tg(isl1*:*GFP)* embryos at the level of r6 and *Tg(isl1*:*GFP)/Tg(isl1*:*cdh2ΔEC-mCherry)vc25* embryos at the level of r4. FBMNs (α-GFP, green) in control *Tg(isl1*:*GFP)* embryos occupy a ventral position within r6. Cdh2ΔEC-mCherry-expressing FBMNs (α-mCherry, red) are found ectopically in a dorsal portion within r4. Nuclei are labeled with DAPI (blue). (E,F) Tracings of migratory paths of FBMNs captured from time-lapse images between 20–24 hpf from *Tg(isl1*:*GFP)* and *Tg(isl1*:*GFP)/Tg(isl1*:*cdh2ΔEC-mCherry)vc25* embryos. Each time-lapse lasted 35 minutes with one frame every 5 minutes. Each trace is oriented so that caudal is to the bottom and medial is to the right. Arrowheads indicate the starting point for each cell. (G) Plot of the migratory tracks from start to endpoint shows a highly directional caudal migration of wild-type FBMNs (green arrows) in comparison to the random paths taken by Cdh2ΔEC-mCherry-expressing FBMNs (red arrows). C, caudal; R, rostral; L, lateral; M, medial (H) Quantitation of average distance traveled along the rostral-caudal axis by FBMNs in *Tg(isl1*:*GFP)* and *Tg(isl1*:*GFP)/Tg(isl1*:*cdh2ΔEC-mCherry)vc25* embryos during the time-lapse sequences. (I) Quantitation of average instantaneous speed of FBMN movements in *Tg(isl1*:*GFP)* and *Tg(isl1*:*GFP)/Tg(isl1*:*cdh2ΔEC-mCherry)vc25* embryos. (Mean values ± SD are shown; *p* < 0.05).

In previous studies, Cdh2 was shown to be required for proper axonal pathfinding of spinal cord motorneurons [36]. We therefore examined axonal pathfinding in *Tg(isl1*:*cdh2ΔEC-mCherry)vc25* fish. Analysis of confocal images from lateral views of *vc25Tg* embryos showed no gross abnormality in the peripheral pathfinding of FBMN axons, or the axons of other CBMNs compared with wild-type embryos (Fig 3A and 3B). This suggests that cell autonomous loss of Cdh2 in FBMNs does not affect axon extension or growth cone steering in the periphery but has a specific effect on the positioning of FBMN cell bodies.

Examination of lateral views of *vc25Tg* embryos showed that Cdh2ΔEC-mCherry-expressing FBMN cell bodies that fail to exit r4 lie in an aberrantly dorsal position (Fig 3B). This was confirmed in confocal images of hindbrain cross sections at 48 hpf from wild-type and homozygous *vc25Tg* embryos. First, consistent with the observation that our dominant-negative transgene was only expressed in cranial motor neurons, the development of the neural tube and overall organization of the neuroepithelial cells was normal in *vc25Tg* embryos (Figs 1K and 3C and 3D). Secondly, Cdh2ΔEC-mCherry-expressing FBMN cell bodies, that fail to migrate caudally out of r4, are instead found in an aberrantly apical and dorsal position within r4 as compared to the normal ventral positioning of FBMNs within r6 of wild-type embryos (Fig 3C and 3D). These results show that cell autonomous loss of Cdh2 function within FBMNs leads to ectopic positioning of FBMNs within r4.

To determine the basis for the neuron position changes seen in *Tg(isl1*:*cdh2ΔEC-mCherry)vc25* fish, we recorded timelapse movies of FBMN movement in wild-type and homozygous *vc25Tg* embryos. We recorded short 35-minute movies at one frame every 5 minutes between 18 and 22 hpf and tracked the cell movements of individual FBMNs at each of the 7 timeframes. Cell tracings reveal that FBMNs in wild-type embryos exhibit sustained directed migration in the posterior (caudal) direction, whereas FBMNs in homozygous *vc25Tg* embryos migrate randomly with many directional changes (Fig 3E and 3F). Consistent with these observations, the total caudal displacement of wild-type FBMNS was significantly larger than the overall caudal displacement of Cdh2ΔEC-mCherry-expressing FBMNs (Fig 3G and 3H). Although inactivation of Cdh2 did not inhibit cell motility, we did quantify a small statistically significant decrease in instantaneous speed of FBMNs in *vc25Tg* embryos (Fig 3I). Together, these findings suggest that Cdh2 is not essential in FBMNs for cell motility, but rather helps to coordinate sustained directed caudal migration of FBMNs.

### Cadherin-2 is required for collective FBMN migration

Studies of many cell types that engage in collective migration indicate that collectiveness is borne from cell-cell interactions, and thus coordinated migration stems from interactions with their neighbors [4,37,38]. Our previous work demonstrated that FBMN migration can be characterized as a collective cell migration, in which one FBMN can influence the migration of a neighboring neuron [22]. FBMNs migrate caudally because they engage both PCP-dependent and collective modes of migration. A PCP-deficient FBMN can still be directed caudally because it can respond to cell-cell contact mediated collective migration from a neighboring wild-type FBMNs in chimeric embryos [22,23]. Thus, we sought to determine whether Cadherin-2 is a candidate cell adhesion molecule controlling cell contact-mediated collective migration of FBMNs.

**Fig 4:**
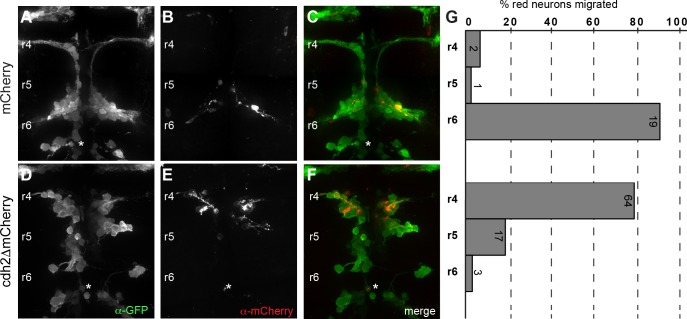
Cadherin-2 is required for collective migration of FBMNs. (A-C) Confocal images showing dorsal views of *Tg(isl1*:*GFP)* embryos at 38 hpf injected with plasmids driving expression of mCherry alone or Cdh2ΔEC-mCherry mosaically in CBMNs. Embryos are labeled with α-GFP (green) and α-mCherry (red). Expression of mCherry alone has no effect on the caudal migration of FBMNs. (D-F) In contrast, FBMNs expressing Cdh2ΔEC-mCherry do not migrate caudally even though neighboring wild-type FBMNs that do not express the transgene migrate appropriately towards r6. (G) Quantitation of the percent of mCherry- or Cdh2ΔEC-mCherry-expressing FBMNs that failed to migrate (r4), migrated partially (r5), or migrated fully (r6). Each histogram corresponds to the condition in the image to its left and numbers indicate the number of FBMNs counted. White asterisk denotes PLL efferent neurons, which differ from r4-derived FBMN populations.

To address this, we used Tol2-mediated transient transgenesis to generate F0 mosaic embryos in which a subset of FBMNs express *cdh2ΔEC-mCherry* under control of the *isl1* promoter (*Tol2-isl1*:*cdh2ΔEC-mCherry-pA-Tol2*) adjacent to non-expressing wild-type FBMNs. We reasoned that if Cdh2 function is required for collective migration, then *cdh2ΔEC-mCherry*-expressing FBMNs would not be influenced or ‘rescued’ in their migration by neighboring wild-type FBMNs. As a control, we expressed *mCherry* mosaically in *Tg(isl1*:*GFP)* embryos. We found that 90.3 ± 11.1% of mCherry-expressing FBMNs migrated normally into r6 in wild-type *Tg(isl1*:*GFP)* embryos (*n* = 22 neurons; 6 embryos) (Fig 4A–4C). In contrast, we found that 78.1 ± 17.6% of Cdh2ΔEC-mCherry-expressing FBMNs remained in r4, with 18.6% remaining in r5, and only 3.3% reaching r6 (n = 84 neurons in 8 embryos) (Fig 4D–4F). This defect in caudal migration is not improved compared to that seen in stable *Tg(isl1*:*cdh2ΔEC-mCherry)vc25* embryos alone, suggesting that the presence of neighboring wild-type FBMNs has no influence on the caudal migration of Cdh2ΔEC-mCherry-expressing FBMNs (Figs 2G–2I and 4D–4F). Interestingly, we noted that wild-type FBMNs do not migrate as efficiently in embryos expressing Cdh2ΔEC-mCherry mosaically, as compared to control embryos (Fig 4F). This suggests that having a subset of FBMNs that cannot engage in collective migration impacts the overall caudal directionality of the group of wild-type FBMNs. Taken together, these results demonstrate that Cdh2 is required for FBMN-to-FBMN cell contact mediated collective migration of FBMNs.

### Neuron-specific inactivation of Cadherin-2 leads to aberrant positioning of other neuronal populations

**Fig 5:**
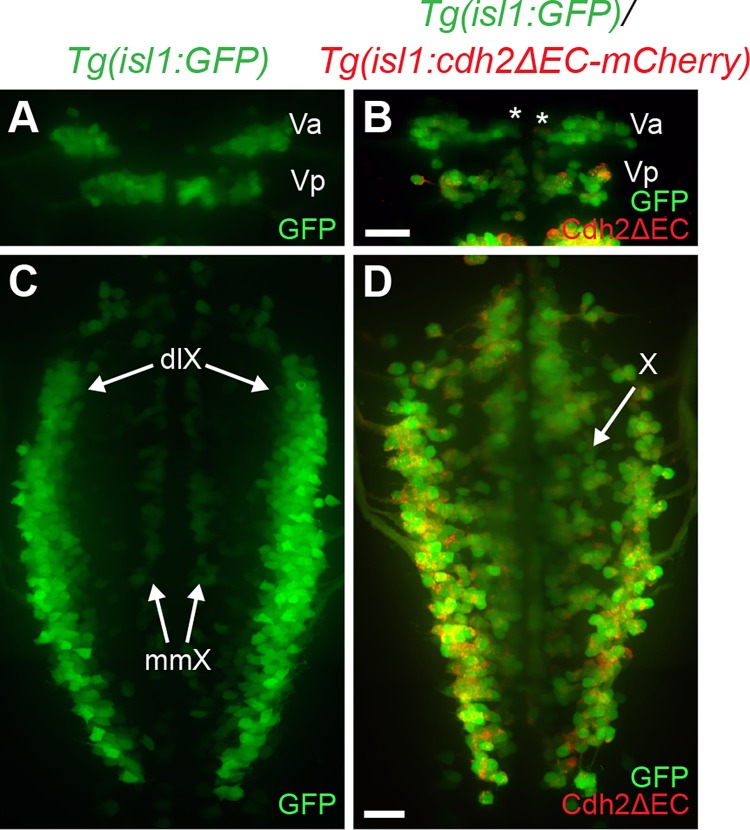
Expression of dominant-negative Cadherin-2 in trigeminal and vagus branchiomotor neurons leads to aberrant neuron positioning. (A) Dorsal view of live *Tg(isl1*:*GFP)* embryo at 48hpf shows positioning of anterior and posterior clusters of trigeminal neurons (Va,Vp) found in r2 and r3, respectively. Note the lateral positioning of the Va cluster of trigeminal motor neurons at 48 hpf. (B) Dorsal view of live *Tg(isl1*:*GFP)/Tg(isl1*:*cdh2ΔEC-mCherry)vc25* embryo at 48 hpf shows that trigeminal neurons (Va; asterisk) remain in a medial location. *Green* is the GFP signal, whereas *red* is the Cdh2ΔEC-mCherry signal. (C) Dorsal view of live *Tg(isl1*:*GFP)* embryo at 48hpf shows correct positioning of vagus motor neurons in dorsolateral motor nucleus (dlX) and medial motor nucleus (mmX). (D) Dorsal view of live *Tg(isl1*:*GFP)/Tg(isl1*:*cdh2ΔEC-mCherry)vc25* embryo shows that vagus neurons (X) do not migrate and coalesce into discrete dorsolateral nuclei. Scale Bars = 20 μm.

To determine whether expression of dominant-negative Cdh2 affected the migration of other neuronal cell types, we examined the cell soma positioning of other CBMNs in *Tg(isl1*:*cdh2ΔEC-mCherry)vc25* embryos. In wild-type embryos, trigeminal branchiomotor neurons are born in r2 and r3 and make up the anterior (Va) and posterior (Vp) trigeminal motor nuclei, respectively [13]. The trigeminal motor neuron cell bodies are located medially early in development and then migrate laterally to their final positions [13]. At 48 hpf, trigeminal neurons in r2 can be seen undergoing their lateral migration in wild-type embryos but not in *Tg(isl1*:*cdh2ΔEC-mCherry)vc25* embryos (Fig 5A and 5B). Vagus motor neurons are born ventrally and undergo a dorsolateral tangential migration to reside in two dorsal columns known as the dorsolateral motor nucleus (dlX) of the vagus nerve as well as two smaller medial columns called the medial motor nucleus (mmX) of the vagus nerve (Fig 5D). However, vagus motor neurons in *vc25Tg* embryos fail to coalesce into distinct dorsal and medial motor nuclei, and instead are widely distributed across the mediolateral aspect of the caudal hindbrain (Fig 5C and 5D). Interestingly, the pathfinding of trigeminal and vagal axons in the periphery is grossly normal in *Tg(isl1*:*cdh2ΔEC-mCherry)vc25* embryos compared to wild-type controls, indicating that neuron-specific inactivation of Cdh2 leads to a specific effect on cell body positioning of CBMNs (Fig 2A and 2B). We also found that inactivation of Cdh2 lead to a defect in the positioning of hindbrain efferent neurons. PLL efferent neurons that innervate neuromasts in the posterior lateral line are born in r6 and migrate caudally into r7, while their axons exit the hindbrain at r6 [13,30,39]. In *Tg(isl1*:*cdh2ΔEC-mCherry)vc25* embryos, many PLL efferents display a partial migration defect with some PLL efferent cell bodies remaining in r6 (Figs 1M–1S and 2A–2I). Taken together, Cdh2 function is required cell autonomously within multiple cranial motor neuron populations for proper neuron migration.

## Discussion

In this report, we define a cell autonomous role for Cadherin-2 in mediating the collective migration of FBMNs. We first generated stable transgenic lines expressing dominant-negative Cdh2 specifically within FBMNS and not the surrounding neuroepithelial cell environment. We show that Cadherin-2 is required cell autonomously in FBMNs for sustained migration in the caudal direction. We found that expression of dominant-negative Cdh2 in FBMNs leads to random, non-directed migration, suggesting that Cdh2 is not required for motility, but promotes a coordinated caudal trajectory of FBMNs. Using mosaic analysis, we demonstrate that dominant-negative Cdh2-expressing FBMNs are unable to be ‘rescued’ by neighboring wild-type neurons, supporting a model in which the collective cell behaviors of FBMNs are driven by Cdh2-based neuron-to-neuron interactions.

### Cadherin-2 controls caudal directionality

During neural development, Cadherin-2 is required for the proper morphogenesis of the neural tube [6,7,25]. The severe neural tube formation defects seen in Cdh2 knockouts or mutants makes it difficult to assess the function of Cdh2 in individual neurons at later stages of CNS development. In zebrafish, transplant experiments to assess *cdh2* mutant cell behavior in a wild-type host environment are difficult since *cdh2*-deficient cells do not integrate into ventral neural tube positions due to adhesive differences [6,25]. In this study, we bypass these problems by disrupting Cadherin-2 function specifically in FBMNs via expression of a dominant-negative form of Cadherin-2 under control of *islet1* enhancer elements that drive expression in cranial branchiomotor neurons. As our transgene was not expressed in the surrounding neuroepithelial cells, we were able to examine the cell autonomous role of Cadherin-2 in FBMN migration without any confounding issues of neural tube morphogenesis. We found that inactivation of Cdh2 in FBMNs leads to defective caudal migration. Having several lines of stable transgenic fish (*vc23*, *vc25*) that displayed similar FBMN migration defects suggests that our observed phenotypes are due to functional Cdh2 inactivation and not positional effects of transgene insertion. The difference in phenotype severity across our lines underlies the importance of strong transgene expression when using a dominant-negative approach and indicates that partial defects in caudal migration, such as those seen in hemizygous *Tg(isl1*:*cdh2ΔEC-mCherry)* embryos represent a hypomorphic phenotype due to incomplete inactivation of Cdh2 function. In homozygous *vc25Tg* embryos, our highest expressing line, lack of Cdh2 function does not affect cell motility per se, but does result in a lack of directionality in FBMN cell soma movements. In the absence of sustained caudally directed migration, FBMNs often make ectopic apical and dorsal movements between neuroepithelial cells within r4 in *Tg(isl1*:*cdh2ΔEC-mCherry)* fish. It was previously suggested that FBMNs are excluded from entering the dorsal hindbrain due to neuroepithelial cell cohesion that stems from Cdh2-mediated homotypic neuroepithelial cell adhesion that ensures that FBMNs migrate along the ventral aspect of the hindbrain [26]. Our observation that dominant-negative Cdh2-expressing FBMNs move dorsally despite normal Cdh2 function in neuroepithelial cells suggests that Cdh2-mediated neuroepithelial cell cohesion is not responsible for excluding FBMNs from the dorsal neuroepithelium. Interestingly, similar ectopic dorsally positioned FBMNs that fail to migrate caudally have been observed in embryos with a mutation in the PCP component Prickle1b (*pk1b*^*fh122*^), where the surrounding neuroepithelial cells are planar polarized with normal structural integrity [17,18,22,40]. Thus, in both cases, inactivation of Cdh2 and depletion of Pk1b, FBMNs migrate randomly and dorsally even when Cdh2-based neuroepithelial cell cohesion is unaffected. Ectopic dorsal positioning of FBMNs in the absence of Cdh2 function appears to be a consequence of a loss of directionality, and contributes to the overall defect in caudal migration. We are, however, unable to rule out the possibility that Cdh2-based neuroepithelial cell-to-FBMN heterotypic cell interactions provide a molecular signal to prevent ectopic apico-dorsal cell movement of FBMNs. Taken together, our findings suggest that Cdh2 acts cell autonomously to promote directional migration of FBMNs.

Despite a reduction in sustained caudal migration when Cdh2 is inactivated, we observed a small proportion of FBMNs that migrated out of r4 into r5 before stalling; a result previously reported in *cdh2* mutants and morphants [26,27]. Our results clarify that the limited caudal migration into r5 is not a byproduct of defective neural tube morphogenesis, but rather suggests that FBMNs retain a limited capacity for caudal migration in the absence of Cdh2 function. Previous studies occasionally observed a small number of FBMNs (‘escapers’) that migrated into r6 in *cdh2* mutants and morphants [26,27]. Similarly, we infrequently found escapers in r6 in some homozygous *vc25Tg* embryos. Our observation that 59% of homozygous *vc25Tg* embryos have at least one escaper neuron agree well with previous estimates of escapers in *cdh2* morphants (61.5% of embryos)[27]. These authors described the leading or pioneer FBMN as having a crucial role in promoting the caudal migration of later-born follower neurons, at least into r5, since laser ablation of the pioneer neuron or its trailing axon impairs follower FBMN migration [27]. These authors attributed the presence of escaper neurons in r6 of Cdh2-depleted embryos as pioneer neurons that migrate in a Cdh2-independent manner, suggesting that pioneer neurons have intrinsically different migratory properties than follower neurons. However, our observation that most *vc25Tg* embryos (86%) have either no escapers (41%) or escapers on only one side of the embryo (45%) is not consistent with a model in which each embryo should have at least one pioneer neuron (per hemi-segment) that navigates caudally without the need for Cdh2 function. We suggest that Cdh2 is one of multiple guidance cues that FBMNs utilize to undergo sustained directed caudal migration. Thus, both leader and follower FBMNs must integrate Cdh2 function with other external guidance cues for robust caudal migration. Elimination of one guidance mechanism, such as Cdh2 neuron-to-neuron interactions, severely limits the overall effectiveness of caudal migration. Interestingly, partial migration phenotypes and ‘escaper’ neurons are not observed in embryos with mutations in PCP components[16,18–21,41], in which FBMNs completely fail to exit r4, despite the observation that PCP mutant FBMNs are motile and migrate randomly within r4 [19,40]. This suggests that Cdh2-deficient FBMNs may rely on PCP-dependent migration to undergo limited and sparing migration out of r4.

### Cadherin-2 controls collective migration

FBMNs exhibit two modes of migration. First, FBMN migration involves interactions between migrating neurons and the surrounding neuroepithelium, an interaction that requires the function of PCP components both within FBMNs (cell-autonomously) and in the surrounding neuroepithelial cells (non cell-autonomously) [22,23]. For instance, wild-type FBMNs fail to migrate through a PCP-deficient neuroepithelial environment [16,19,21]. On the other hand, when all FBMNs are PCP-deficient, they are unable to migrate caudally even when the environment is wild-type [18,22,23]. Second, FBMNs engage in a collective cell migration that involves FBMN-to-FBMN interactions. In chimeric embryos generated by cell transplantation, a wild-type FBMN can induce caudal directional movements from a neighboring PCP-deficient FBMN [22]. This influence of a wild-type FBMN on the directional migration of another neuron characterizes this as a collective cell migration that involves neuron-to-neuron interactions but does not require cell autonomous PCP function in the rescued neuron. In previous studies assessing migratory phenotypes in chimeras, up to 40% of PCP-deficient neurons migrate caudally if adjacent to neighboring wild-type FBMNs [22]. It is striking then that dominant-negative Cdh2-expressing FBMNs completely fail to be “rescued” by neighboring wild-type FBMNs in mosaic embryos in this study. Our observations are consistent with a model in which Cdh2-mediated homotypic FBMN-to-FBMN cell interactions are the basis for collective cell migration in FBMNs.

What is the nature of the Cdh2-based neuron-to-neuron cell contact that drives collective cell behavior in FBMNs? As described above, the first FBMN to exit r4 represents the pioneer neuron that trails its axon behind it as it migrates caudally [27]. Follower FBMNs are proposed to migrate using the pioneer axon as a substrate for adhesion and migration [27]. In this scenario, Cdh2 may function to control attachment of follower neurons to pioneer axons and promote caudal migration along a preferred substrate. This could explain how PCP-deficient cells are ‘rescued’ by wild-type FBMNs, since ‘follower’ PCP-mutant FBMNs could still make use of Cdh2-mediated adhesion to migrate along the wild-type pioneer axon substrate to move in the caudal direction. In this case, cell-cell interactions would represent soma-to-axon adhesions between follower and pioneer FBMNs. This is reminiscent of a role for Cadherin-2 in radial glial fiber-dependent migration of cortical neurons, where Cdh2 mediates tight attachment of locomoting neurons and radial glial fibers [8,9]. An alternative possibility is that caudal directionality is obtained through Cdh2-based soma-to-soma cell contact between FBMNs. In this scenario, Cdh2-mediated cell contact is required for movement and that cell polarity and directionality are acquired as a consequence of soma-to-soma contact. Both neural crest cells and mesendodermal cells migrate as single cells that utilize transient Cdh2-based cell-to-cell contacts to coordinate directionality of movement [38,42,43]. Future studies using high-resolution time-lapse imaging will help resolve the precise nature of Cdh2-mediated neuron-to-neuron interactions that drive collective FBMN migration.

## Supporting Information

S1 Fig Cadherin-2 is required cell autonomously for caudal migration of FBMNs. (A-I) Whole-mount immunocytochemistry showing dorsal views of *Tg(isl1*:*GFP)* (A-C) and *Tg(isl1*:*cdh2ΔEC-mCherry)vc23* transgenic embryos (D-I) at 38 hpf embryos. Embryos are labeled with α-GFP (green) (A,D,G) and α-mCherry (red) (B,E,H) antibodies. (A-C) Wild-type *Tg(isl1*:*GFP)* embryos with FBMNs fully migrated into r6. (D-I) Defective caudal migration of FBMNs in *Tg(isl1*:*GFP)/Tg(isl1*:*cdh2ΔEC-mCherry)vc23* embryos carrying one copy of the transgene (hemizygous) or two copies (homozygous). (J) Histograms indicate the percent of FBMNS at 38 hpf that failed to migrate (r4), migrated partially (r5), or migrated fully (r6). Each histogram corresponds to the genetic condition in the image to its left and numbers indicate the number of FBMNs counted.
(TIF)

S2 Fig Migration of ‘escaper’ neurons in homozygous *Tg(isl1*:*cdh2ΔEC-mCherry)vc25* embryos. (A-E) Confocal micrographs of dorsal views of homozygous *Tg(isl1*:*GFP)/Tg(isl1*:*cdh2ΔEC-mCherry)vc25* embryos at 38 hpf. Embryos were labeled with α-GFP (green) and α-mCherry (red). Representative images of homozygous *vc25Tg* embryos that shows the majority of FBMNs fail to exit r4/r5 with or without a rare ‘escaper’ FBMN that migrates into r6 (arrows). (A) An embryo with no ‘escaper’ neurons present in r6 on either side of the midline (0/0). (B). An embryo with one ‘escaper’ neuron present in r6 on one side of the embryo, with no ‘escapers’ on the contralateral side (1/0). (C) An embryo with two ‘escaper’ FBMNs present in r6 on one side of the embryo and no ‘escapers’ on the contralateral side (2/0). (D) An embryo with one ‘escaper’ neuron present in r6 on both sides of the embryo (1/1). (E) An embryo with one ‘escaper’ neuron present in r6 on one side of the embryo and two ‘escaper’ FBMNs present on the contralateral side (1/2). (F) Histogram reflects the percentage of homozygous *Tg(isl1*:*cdh2ΔEC-mCherry)vc25* embryos with each ‘escaper’ condition. (G-I) Confocal micrographs of immunostained embryos showing high magnification dorsal views of *Tg(isl1*:*GFP)/Tg(isl1*:*cdh2ΔEC-mCherry)vc25* embryo at 38 hpf. White arrow shows ‘escaper’ neuron that expresses both *isl1*:*GFP* (green) and *isl1*:*cdh2ΔEC-mCherry* (red) transgenes, despite its presence in r6. White asterisk denotes r6-derived PLL efferent neurons, which differ from r4-derived FBMN populations.
(TIF)

S3 Fig Defect in caudal migration is not due to a delay in cell movements in *Tg(isl1*:*cdh2ΔEC-mCherry)vc25* transgenic embryos. (A-B) Whole-mount immunocytochemistry showing dorsal view of wild-type *Tg(isl1*:*GFP)* (A) and *Tg(isl1*:*cdh2ΔEC-mCherry)vc25* transgenic embryos (B) at 48 hpf. Embryos are labeled with α-GFP (green) (A and B) and α-mCherry (red) (B) antibodies. (A) Wild-type *Tg(isl1*:*GFP)* embryos with FBMNs fully migrated into r6. (B) There is a dramatic defect in caudal migration of FBMNs in homozygous *Tg(isl1*:*cdh2ΔEC-mCherry)vc25* embryos at 48 hpf, when FBMN migration is normally complete. (C) Histogram indicates the percent of FBMNS at 48 hpf that failed to migrate (r4), migrated partially (r5), or migrated fully (r6). Each histogram corresponds to the genetic condition in the image to its left and numbers indicate the number of FBMNs counted. White asterisk denotes r6-derived PLL efferent neurons, which differ from r4-derived FBMN populations.
(TIF)

S1 Movie Time-lapse of FBMN migration in *Tg(isl1*:*GFP)* embryos. The images are maximum projections of z-slices. Time between frames is 5 minutes.
(AVI)

S2 Movie Time-lapse of FBMN migration in *Tg(isl1*:*GFP)/Tg(isl1*:*cdh2ΔEC-mCherry)vc25* embryos. The images are maximum projections of z-slices. Time between frames is 5 minutes.
(AVI)
