# Supplementary figures and images for: Cadherin-2 Is Required Cell Autonomously for Collective Migration of Facial Branchiomotor Neurons
Source: PLoS One. 2016 Oct 7;11(10):e0164433. doi: 10.1371/journal.pone.0164433 (PMC5055392; doi:10.1371/journal.pone.0164433)

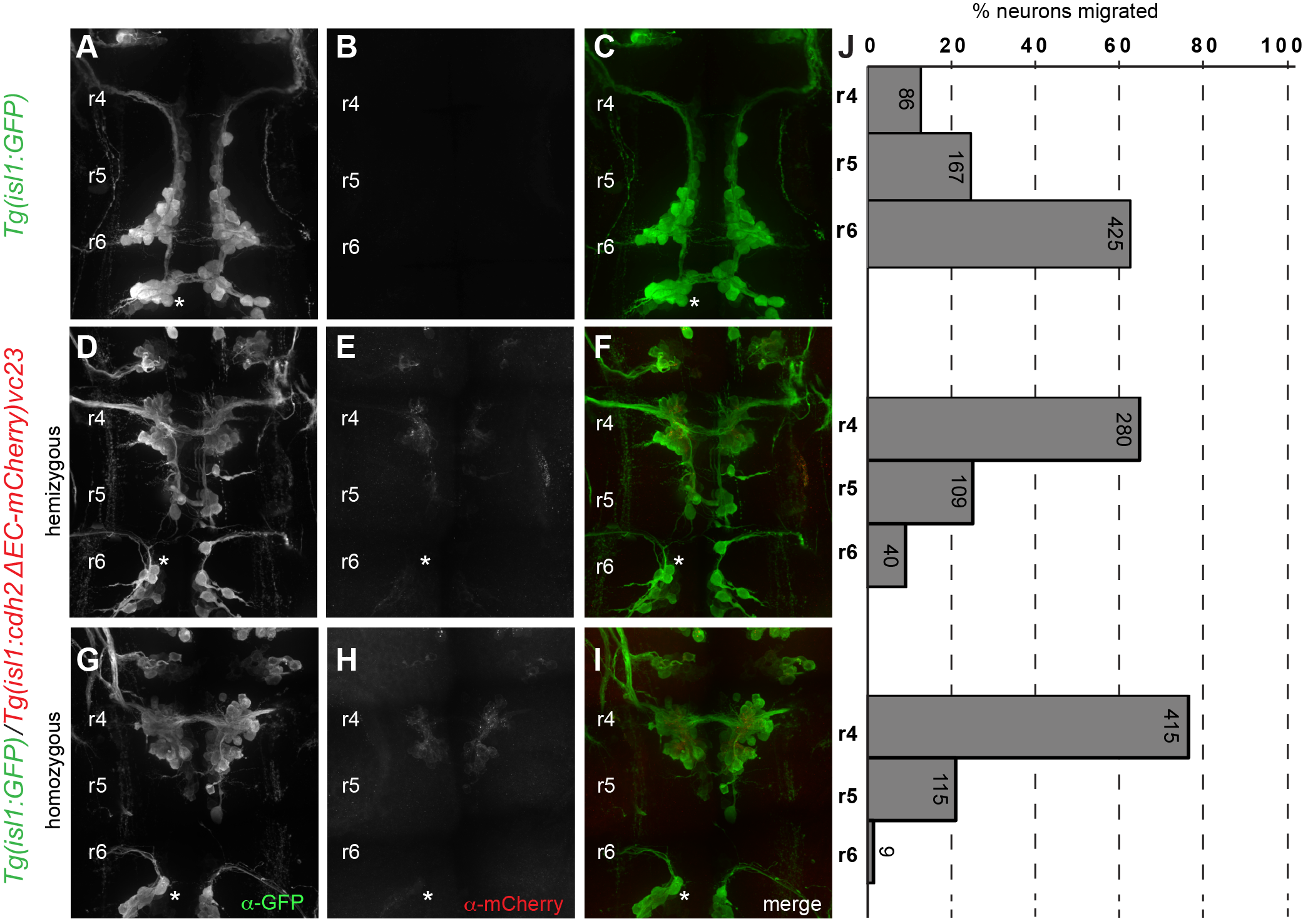

Supplement: S1 Fig — (A-I) Whole-mount immunocytochemistry showing dorsal views of Tg(isl1:GFP) (A-C) and Tg(isl1:cdh2ΔEC-mCherry)vc23 transgenic embryos (D-I) at 38 hpf embryos. Embryos are labeled with α-GFP (green) (A,D,G) and α-mCherry (red) (B,E,H) antibodies. (A-C) Wild-type Tg(isl1:GFP) embryos with FBMNs fully migrated into r6. (D-I) Defective caudal migration of FBMNs in Tg(isl1:GFP)/Tg(isl1:cdh2ΔEC-mCherry)vc23 embryos carrying one copy of the transgene (hemizygous) or two copies (homozygous). (J) Histograms indicate the percent of FBMNS at 38 hpf that failed to migrate (r4), migrated partially (r5), or migrated fully (r6). Each histogram corresponds to the genetic condition in the image to its left and numbers indicate the number of FBMNs counted. (TIF) [file pone.0164433.s001.tif]

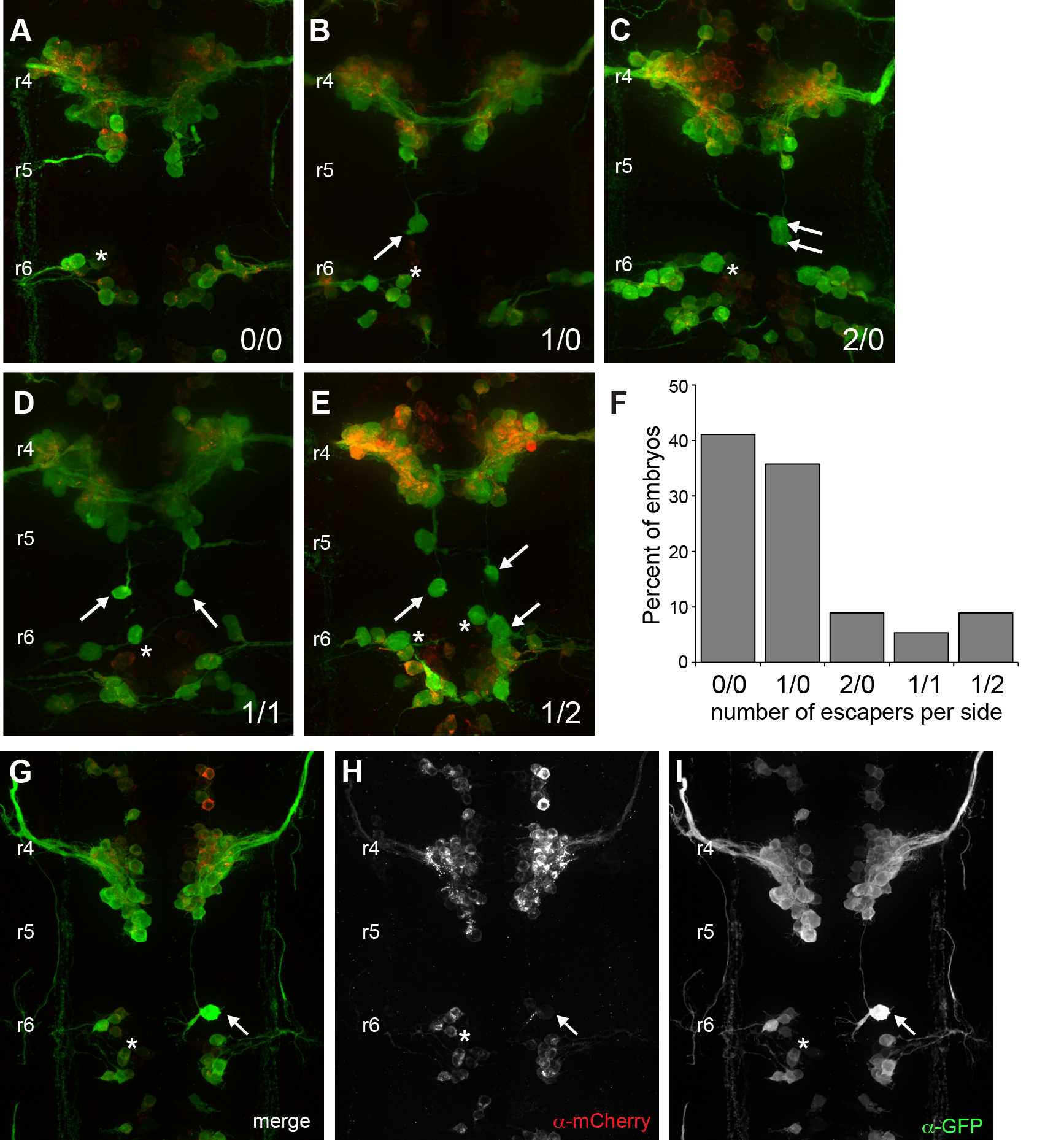

Supplement: S2 Fig — (A-E) Confocal micrographs of dorsal views of homozygous Tg(isl1:GFP)/Tg(isl1:cdh2ΔEC-mCherry)vc25 embryos at 38 hpf. Embryos were labeled with α-GFP (green) and α-mCherry (red). Representative images of homozygous vc25Tg embryos that shows the majority of FBMNs fail to exit r4/r5 with or without a rare ‘escaper’ FBMN that migrates into r6 (arrows). (A) An embryo with no ‘escaper’ neurons present in r6 on either side of the midline (0/0). (B). An embryo with one ‘escaper’ neuron present in r6 on one side of the embryo, with no ‘escapers’ on the contralateral side (1/0). (C) An embryo with two ‘escaper’ FBMNs present in r6 on one side of the embryo and no ‘escapers’ on the contralateral side (2/0). (D) An embryo with one ‘escaper’ neuron present in r6 on both sides of the embryo (1/1). (E) An embryo with one ‘escaper’ neuron present in r6 on one side of the embryo and two ‘escaper’ FBMNs present on the contralateral side (1/2). (F) Histogram reflects the percentage of homozygous Tg(isl1:cdh2ΔEC-mCherry)vc25 embryos with each ‘escaper’ condition. (G-I) Confocal micrographs of immunostained embryos showing high magnification dorsal views of Tg(isl1:GFP)/Tg(isl1:cdh2ΔEC-mCherry)vc25 embryo at 38 hpf. White arrow shows ‘escaper’ neuron that expresses both isl1:GFP (green) and isl1:cdh2ΔEC-mCherry (red) transgenes, despite its presence in r6. White asterisk denotes r6-derived PLL efferent neurons, which differ from r4-derived FBMN populations. (TIF) [file pone.0164433.s002.tif]

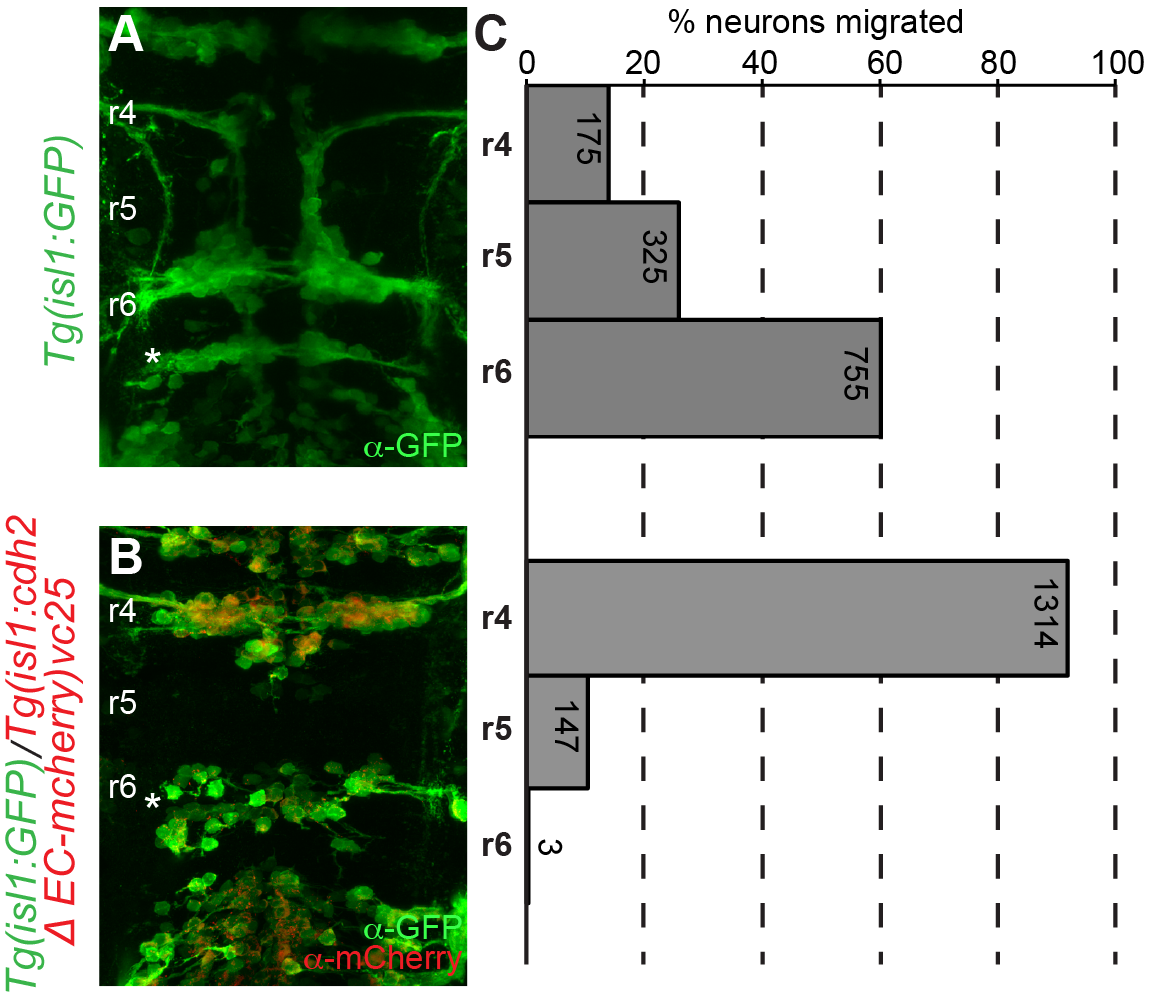

Supplement: S3 Fig — (A-B) Whole-mount immunocytochemistry showing dorsal view of wild-type Tg(isl1:GFP) (A) and Tg(isl1:cdh2ΔEC-mCherry)vc25 transgenic embryos (B) at 48 hpf. Embryos are labeled with α-GFP (green) (A and B) and α-mCherry (red) (B) antibodies. (A) Wild-type Tg(isl1:GFP) embryos with FBMNs fully migrated into r6. (B) There is a dramatic defect in caudal migration of FBMNs in homozygous Tg(isl1:cdh2ΔEC-mCherry)vc25 embryos at 48 hpf, when FBMN migration is normally complete. (C) Histogram indicates the percent of FBMNS at 48 hpf that failed to migrate (r4), migrated partially (r5), or migrated fully (r6). Each histogram corresponds to the genetic condition in the image to its left and numbers indicate the number of FBMNs counted. White asterisk denotes r6-derived PLL efferent neurons, which differ from r4-derived FBMN populations. (TIF) [file pone.0164433.s003.tif]
